# Effects of wall suction/blowing on two-dimensional flow past a confined square cylinder

**DOI:** 10.1186/s40064-016-2666-7

**Published:** 2016-07-04

**Authors:** Wei Zhang, Yanqun Jiang, Lang Li, Guoping Chen

**Affiliations:** School of Civil Engineering and Architecture, Southwest University of Science and Technology, Mianyang, 621010 Sichuan People’s Republic of China; Department of Modern Mechanics, University of Science and Technology of China, Hefei, 230027 Anhui People’s Republic of China; Department of Mathematics, Southwest University of Science and Technology, Mianyang, 621010 Sichuan People’s Republic of China

**Keywords:** Square cylinder, Channel, Suction, Blowing, Bifurcation

## Abstract

A numerical simulation is conducted to study the laminar flow past a square cylinder confined in a channel (the ratio of side length of the square to channel width is fixed at 1/4) subjected to a locally uniform blowing/suction speed placed at the top and bottom channel walls. Governing equations with boundary conditions are resolved using a finite volume method in pressure–velocity formulation. The flow patterns relevant to the critical spacing values are investigated. Numerical results show that wall blowing has a stabilizing effect on the flow, and the corresponding critical Reynolds number increases monotonically with increasing blowing velocity. Remarkably, steady asymmetric solutions and hysteretic mode transitions exist in a certain range of parameters (Reynolds number and suction speed) in the case of suction.

## Background

Fluid flow past a confined or unconfined square cylinder is a model problem of fundamental interest because it affects a number of practical engineering applications, such as building aerodynamics and cooling of electronics. Many previous studies are associated with the simulation of 2D flow around bluff obstacles (Guo et al. 2003; Turki et al. 2003; Galletti et al. 2004; Camarri and Giannetti 2007; Rashidi and Esfahani 2015). 2D incompressible flow past a cylinder is known to be steady, laminar, and symmetric at sufficiently low flow rates. This steady flow loses stability at a critical Reynolds number beyond which vortices are formed and shed alternately behind the cylinder, thereby causing a periodic flow in its wake region. This periodic vortex street causes fluctuating lift and drag forces, which are major factors in most flow systems in engineering and industrial applications, e.g., deterioration of vehicle performance and fatigue of mechanical structures. Thus, eliminating or restraining fluctuating forces and vortex shedding from such bodies is necessary.

Suction/blowing method, one of the most effective active flow-control methods, has been widely used in many aspects, such as bluff body (Mathelin and Maitre 2009; Weller et al. 2009; Zheng and Zhang 2012; Shtendel and Seifert 2014; Chen et al. 2013; Muralidharan et al. 2013; Boujo and Gallaire 2014; Sohankar et al. 2015; Layek et al. 2008a), backward-facing step (Kaiktsis and Monkewitz 2003), symmetric sudden expanded channel  (Layek et al. 2008b), and plane Poiseuille flow (Gao and Lu 2006). In recent years, several researchers have studied the effect of suction/blowing on flow past the bluff body. For example, Mathelin and Maitre (2009) constructed a robust proper orthogonal decomposition basis used on a reduced model to determine the optimal control law for reducing body drag by blowing/suction at the circular cylinder surface. Weller et al. (2009) applied a low-order model to determine a feedback control set by placing blowing and suction actuators on the square cylinder to reduce flow unsteadiness of the bluff body wake at Re = 150. Zheng and Zhang (2012) investigated the performance and mechanism of suction control on drag reduction for a high-rise building. Shtendel and Seifert (2014) conducted experiments on active flow control to control flow around a circular cylinder at transitional Reynolds number, for drag reduction and wake stabilization by applying the combined steady suction and pulsed blowing in close proximity. Chen et al. (2013) conducted an experimental investigation to mitigate vortex-induced vibration of a circular cylinder by using a steady suction flow method. Muralidharan et al. (2013) numerically investigated vortex structures behind a flexibly mounted cylinder and designed a three actuator system in the form of suction and blowing slots positioned on the cylinder surface to suppress vortex-induced oscillations. Boujo and Gallaire (2014) applied linear sensitivity analysis to the 2D steady flow past a circular cylinder in both the subcritical and supercritical regimes and designed control configurations that can reduce recirculation length based on sensitivity information in particular fluid suction at the cylinder wall. Sohankar et al. (2015) investigated the effects of uniform suction and blowing positioned on three different surfaces of a square cylinder on vortex shedding, wake structure, and heat transfer; and found that to achieve optimum configuration, lift and drag fluctuations decreased and maximum reduction on the drag force was 72 % for Re = 150.

Previous investigations on suction/blowing control mainly focused on the unbounded bluff body. When the bluff body is confined in a plane channel, the nature and stability of the resulting flow differ significantly because of the blockage effect induced by the stationary walls of the plane channel. Therefore, suction/blowing positioned on channel walls is an appropriate control method for the flow past a confined square cylinder. To the best of our knowledge, this method has been examined only in the work of Layek et al. (2008a, b), where vortex shedding is argued to be suppressed with the application of local blowing or suction. However, Layek et al. (2008a, b) only considered the flow control for two typical suction/blowing speeds $(V_{s} /V_{b} = 0.1,0.2)$ at a fixed Reynolds number (Re = 600). Therefore, the main objective of the present study is to investigate the effects of wall suction or blowing on the wake of a symmetrically confined square cylinder for a wide range of parameters. This study may help determine the flow bifurcation curves in the Reynolds number-suction/blowing velocity plane.

## Flow configuration and numerical tools

**Fig. 1:**
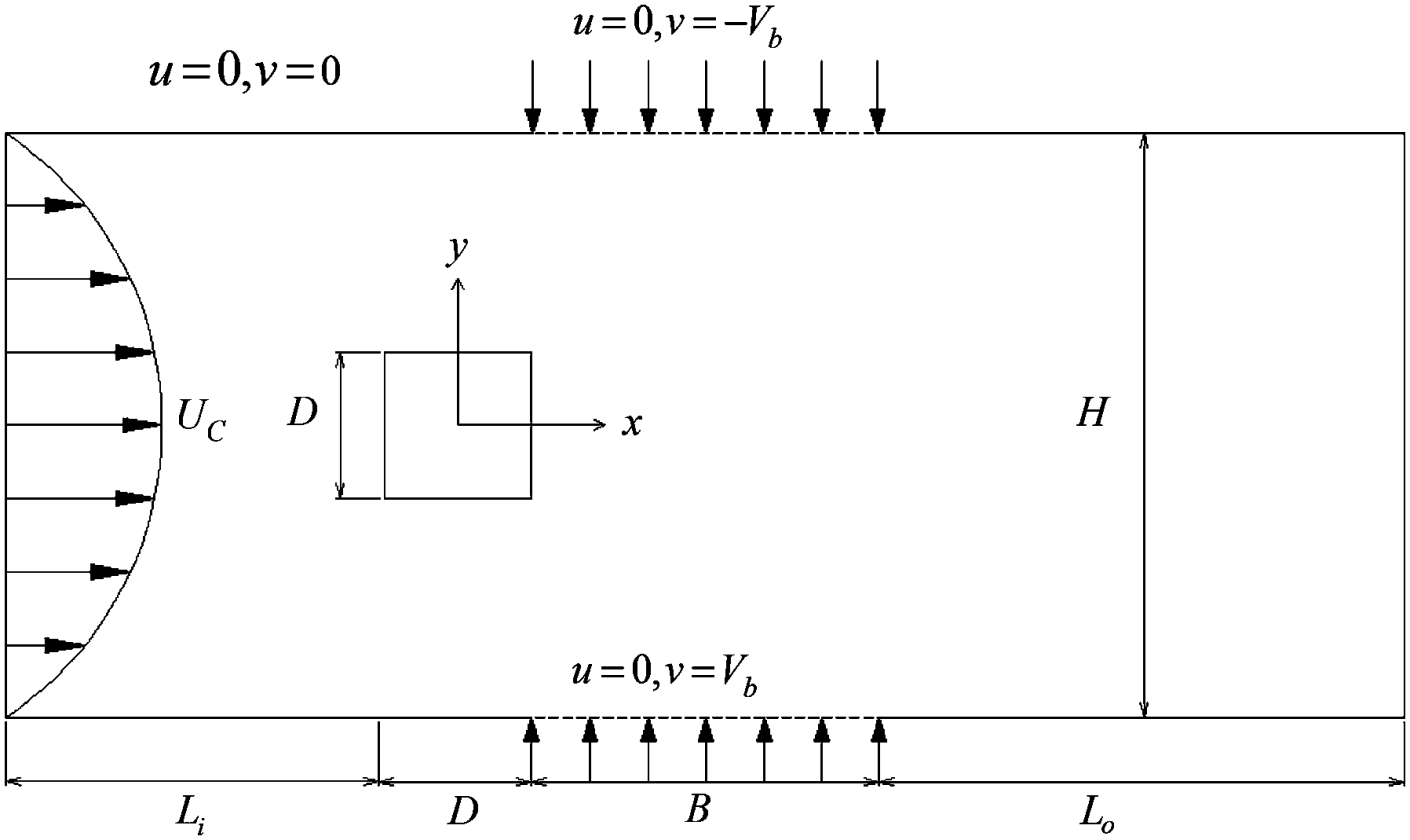
Configuration and coordinates (not to scale) for blowing. In the case of suction, velocities normal to the wall are reversed

We consider a 2D incompressible flow over an infinitely long square cylinder symmetrically confined by two parallel walls. A steady suction or blowing is imposed in the direction perpendicular to the main flow through a porous portion of the channel walls. The porous portion starts at a typical point on each channel wall at which the square cylinder ends and extends horizontally over 4*D* along the wall, where *D* is the side length of the square. A Poiseuille profile with maximum centerline velocity $U_{C}$ is imposed at the inlet boundary and a pressure outlet condition $p = 0$ is applied on the outflow boundary. No-slip boundary conditions are imposed on the surface of the square cylinder and along the channel walls except at the porous part of the walls. The schematic of the flow configuration together with the notation is sketched in Fig. 1. The computational domain is defined as $L_{i} = 11D,\,H = B = 4D,$ and $L_{o} = 32D,$ where *H* is the height of the channel.

We denote *D* and $U_{C}$ as the representative length and velocity scales, respectively. Blockage ratio ($\beta$) and Reynolds number (Re) are defined as $\beta = D /H$ and $\text{Re} = U_{C} D /\nu ,$ respectively, where $\nu$ is the kinematic viscosity of the fluid. In the present study, the following parameter ranges are defined:

1. The range of Re is considered as follows: (a) the flow is 2D and (b) the incoming Poiseuille flow is stable. Thus, the range of Re is limited to $\text{Re} \le 350.$
2. The range of suction/blowing velocity which is made nondimensional with $U_{C}$ is limited to $V_{s} \le 0.4,V_{b} \le 0.4.$
3. Specifically, blockage ratio $\beta$ is fixed at 1/4.

Flow field is obtained by numerical integration of the 2D incompressible Navier–Stokes equations in the following dimensionless forms:

1 $$\frac{{\partial {\mathbf{u}}}}{\partial t} + ({\mathbf{u}} \cdot \nabla ){\mathbf{u}} = - \nabla p + \frac{1}{\text{Re}}\nabla^{2} {\mathbf{u}},$$

2 $$\nabla \cdot {\mathbf{u}} = 0,$$

which are discretized in space on a rectangular Cartesian mesh by finite volume method and integrated in time using a second-order implicit method. SIMPLEC algorithm is applied to resolve pressure–velocity coupling. QUICK scheme and second-order central differences are used for discretization of convective and diffusion terms of the governing equations, respectively. An inner iterative procedure is used to solve the discretization equations. The iteration process is stopped if all scaled residuals level off and are below 0.00001 for all the variables and the continuity equation. The nondimensional time step is set as $\Delta t = 0.001$ in all simulations.

A stretched square mesh is used in this study, which is refined near the cylinder surface and channel wall, where velocity and pressure rapidly vary. To select the most appropriate mesh that can guarantee low computational costs and good resulting accuracy, a set of simulations with four different grid sizes was performed for the non-porous case of $\text{Re} = 200$ and $\beta = 1 /4.$ These grid sizes are listed in Table 1. The most important parameters are the force coefficient along the streamwise (time-averaged drag coefficient $C_{D}$) and the normalized vortex shedding frequency known as the Strouhal number $St = fD /U_{C}$ (where *f* represents the frequency of vortex shedding computed with a rapid Fourier transform of the time trace of the lift coefficient). A comparison of these parameters is also presented in Table 1. The results for cases 3 and 4 are almost identical and an increase in the number of cells from grids 3 to 4 has minimal influence on the results. The convergent time-averaged drag coefficient value $C_{D}$ is 1.63 and the Strouhal number is 0.183, which are in good agreement with the findings of Turki et al. (2003). Therefore, the grid size of case 3 is selected for the subsequent simulations considered in this study.

**Table 1:** Details of the different grid resolutions

Grid no.	Grid size	$C_{D}$	$St$
1	75 × 470	1.581	0.191
2	80 × 770	1.613	0.187
3	110 × 950	1.631	0.183
4	130 × 1200	1.632	0.183

## Results and discussion

To cover the interesting parameter space, we ran more than 200 simulations. All programs were performed on an eight-processor parallel computer to reduce computation time.

**Fig. 2:**
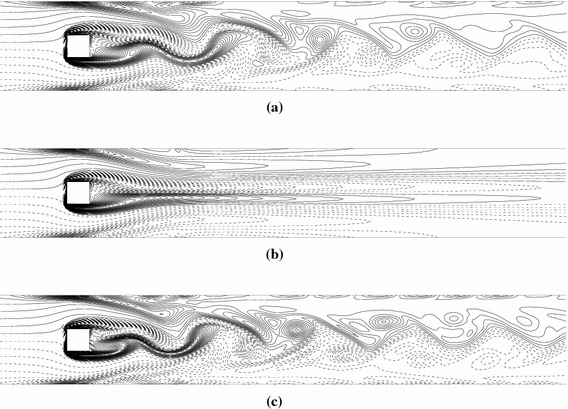
Vorticity contours in case of blowing for different values of **a** $V_{b} = 0.05,\,\text{Re} = 175$, **b** $V_{b} = 0.1,\,\text{Re} = 175$, **c** $V_{b} = 0.1,\,\text{Re} = 250.$ The *solid* and *dashed curves* indicate positive and negative values, respectively

Initially, we present snapshots of the vorticity field, which display the effects of wall blowing on the flow. At a fixed Re of 175, flow is observed to be periodic (Fig. 2a) for $V_{b} = 0.05$ and steady for $V_{b} = 0.1$ (Fig. 2b); thus, an increase in blowing rate suppresses the vortex street. At a fixed blowing velocity of 0.1, flow is steady (Fig. 2b) for $\text{Re} = 175$ and periodic for $\text{Re} = 250$ (Fig. 2c), indicating a destabilizing effect of the Reynolds number.

**Fig. 3:**
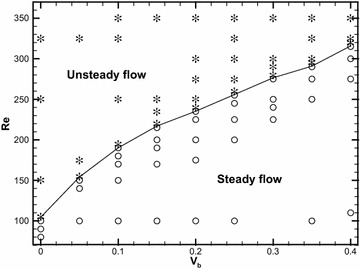
The critical curve in case of blowing in the $\text{Re} \sim V_{b}$ plane separating unsteady and steady flow regimes, based on computed flow states: *stars* unsteady flow and *open circle*, steady flow

To analyze the bifurcation structure of the flow, we take velocity $v$ (i.e., component in the y-direction) at a point located 8D from the rear face of the cylinder, as the representative physical quantity for evaluating the critical parameter. The Cartesian coordinates of the point are x = 8.5D, y = 0. From the relation $(v_{\rm max } - v_{\rm min } ) \propto ({\rm Re} - \text{Re}_{H} )^{1/2}$ (Mizushima and Ino 2008) (where $v_{{\rm max} }$ and $v_{{\rm min} }$ are the maximum and minimum values of monitor velocity $v$ in one period, respectively, and subscript *H* indicates Hopf bifurcation), the critical Reynolds number $\text{Re}_{H}$ beyond which the flow switches from a steady pattern to a periodic flow can be obtained by extrapolation. The region of global flow destabilization as a function of $V_{b}$ is shown in Fig. 3. This figure shows that critical Reynolds number $\text{Re}_{H}$ is a monotonic increasing function of $V_{b} ,$ which indicates a stabilizing effect of blowing. From Fig. 3, flow is periodic above the critical curve and steady below the critical curve. The aforementioned steady flow is symmetric with respect to the central line of the channel and no flow separation on the walls occurs (Figs. 2b, 5a).

**Fig. 4:**
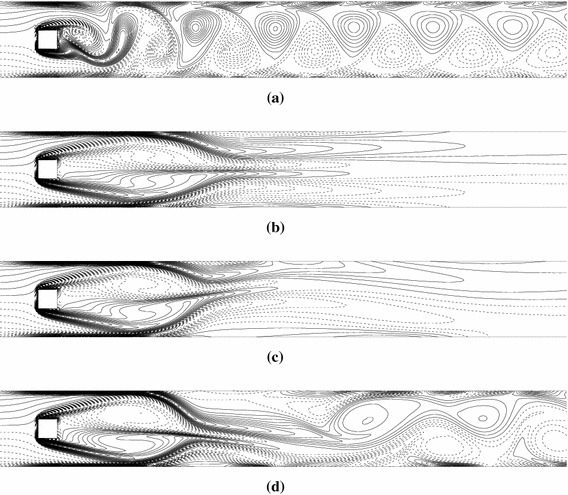
Vorticity contours in case of suction for different values of **a** $V_{s} = 0.1,\,\text{Re} = 175$, **b** $V_{s} = 0.25,\,\text{Re} = 175$, **c** $V_{s} = 0.3,\,\text{Re} = 175$ and **d** $V_{s} = 0.3,\,\text{Re} = 250$

**Fig. 5:**
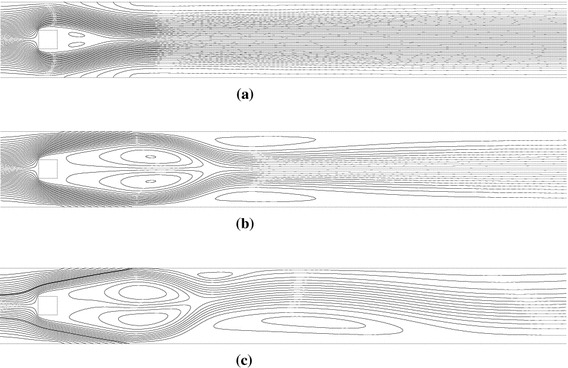
Steady-state streamlines for different values of **a** $V_{b} = 0.1,\,\text{Re} = 175$, **b** $V_{s} = 0.25,\,\text{Re} = 175$ and **c** $V_{s} = 0.3,\,\text{Re} = 175$

When suction velocity is imposed on both channel walls (Fig. 1b), the vortex street can be suppressed by the wall suction, similar to the case of blowing. Results for $\text{Re} = 175$ are presented in Fig. 4, where instantaneous vorticity fields are shown for different values of $V_{s} .$ Clearly, the value of $V_{s}$ has a dramatic effect on flow response. For $V_{s} = 0.1,$ the flow remains periodic (Fig. 4a). As $V_{s}$ is increased to 0.25, the flow bifurcates to a steady and symmetric state (Figs. 4b, 5b). When $V_{s}$ is further increased to 0.3, the wake is steady but asymmetric with respect to the central line of the channel (Fig. 4c). From the vorticity contours (Fig. 4c) and corresponding streamlines (Fig. 5c), the wake behind the cylinder clearly deflects to one of the sides of the channel and the recirculation regions immediately appear not only in the wake of the cylinder but also near the walls. It is noted that the detected steady asymmetric solutions do not always deflect to the upper wall. The flow solutions deflected to the lower wall can also be obtained if different initial conditions are used in numerical simulations. However, due to the symmetry of problem configuration and boundary conditions, these solutions are not distinguished in the context of the present study. When the Reynolds number is further increased to 250, at a fixed suction velocity of 0.3, a periodic oscillatory flow is observed (Fig. 4d). Therefore, an increase in Reynolds number destabilizes the flow.

For all case studies above, the initial condition for the simulation is that no flow is inside the channel at the initial time $(t = 0)$. The chosen initial conditions (motionless fluid) are incompatible with the inlet boundary conditions (parabolic Poiseuille flow). In our computational code, there is an inner iterative procedure to solve the discretization equations at the first time step and the convergence criteria of the scaled residuals for all variables and the continuity equation are set as 0.00001. As time progresses one step, i.e. $t = dt = 0.001,$ the inner flow field is compatible with the boundary condition and all governing equations are satisfied. So the inconsistency exists only for a few steps. For all the test cases, we run the schemes up to the output time $t > 100$ which is far away from the initial disturbance. Therefore, this inconsistency has no effect on the numerical results obtained by our numerical method and our research experiences (such as the results of mesh independence study) have proved this point.

**Fig. 6:**
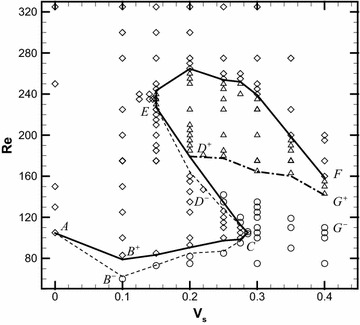
Bifurcation diagram in case of suction in the $\text{Re} \sim V_{s}$ plane: *diamonds*, unsteady flow; *circles*, steady symmetric flow; and *deltas*, steady asymmetric flow

Generally, the influence of the initial condition is ineluctable and different initial condition may result in different flow field. In order to investigate the influence of initial condition on flow pattern, we explore the hysteretic transition for all the blowing/suction speeds when the Reynolds number is varied in two different ways, one being a progressive increase and the other a progressive decrease. Results show that there is no obvious hysteresis effect for the blowing case and hysteresis effect exists in a certain range of parameters (Reynolds number and suction speed) for the suction case. The bifurcation diagram in Fig. 6 summarizes our findings. The different symbols show all the representative cases in this diagram. Diamonds indicate periodic vortex shedding. Circles and deltas correspond to steady symmetric and asymmetric states, respectively.

The critical curves are labeled $AB^{ + } CD^{ + } EF,\,D^{ + } G^{ + } ,\,AB^{ - } CD^{ - } EF$ and $D^{ - } G^{ - }$ where the superscripts + and − denote the critical values in the Re increasing and decreasing ways, respectively. The section of the critical $\text{Re} \sim V_{s}$ curves labeled $AB^{ + } C$ represents a transition curve; by crossing this section in the direction of increasing Reynolds number, the flow loses stability via Hopf bifurcation from a steady symmetric state to a periodic vortex shedding. Section $CD^{ + }$ represents the part of the critical curve; by crossing this section in the direction of increasing $V_{s} ,$ periodic flow restabilizes to a steady symmetric state again. Further increases in the Reynolds number may result in the steady symmetric solution becoming unstable via a pitchfork bifurcation into a steady asymmetric state. The curve of neutral stability for this transition is labeled $D^{ + } G^{ + } .$ Finally, the steady asymmetric solution of the region between the curves *EF* and $D^{ + } G^{ + }$ can become unstable via a Hopf bifurcation into a periodic oscillation state. The transition curve is plotted as *EF*.

The hysteresis is observed in the range between the critical boundaries $AB^{ + } C$ and $AB^{ - } C,\,D^{ + } G^{ + }$ and $D^{ - } G^{ - } ,\,CD^{ + } E$ and $CD^{ - } E.$ The hysteretic transition between steady symmetric flow and periodic oscillatory flow exists in the region between the thick solid line $AB^{ + } C$ and thin dashed line $AB^{ - } C.$ In the Re increasing process the transition from steady symmetric flow to periodic oscillatory flow takes place at a larger Reynolds number than in the Re decreasing process. The hysteretic transition between steady symmetric flow and steady asymmetric flow exists in the region between the thick dashdot line $D^{ + } G^{ + }$ and thin dotted line $D^{ - } G^{ - } .$ The hysteretic transition between periodic oscillatory flow and steady flow exists in a narrow region between the line $CD^{ + } E$ and $CD^{ - } E.$ The upper branch of critical curve (*EF*), obtained with a progressive increase of Re, is coincident with that obtained with a progressive increase of Re, which indicates that no hysteresis exists.

## Conclusions

The effects of uniform suction/blowing speed placed on the top and bottom walls of the channel on the flow past a symmetrically confined square cylinder in a channel have been studied numerically. Various flow patterns and flow bifurcation curves have been obtained for different blowing and suction speeds. Based on the present research, the main conclusions are briefly summarized as follows:

1. Blowing has a stabilizing effect on the flow past a confined cylinder and the corresponding critical Reynolds number increases with the increase in blow speed.
2. Reynolds number has a destabilizing effect on both blowing and suction cases.
3. Steady asymmetric solutions exist in a certain range of parameters in the case of suction.
4. In the case of suction, the critical curves are related not only to the suction speed but also to the changing way of Reynolds number. Hysteretic phenomena of mode exchanges are observed when we increase or decrease Reynolds number continuously at a fixed suction speed.
